# Self-assembling protein nanoparticles and virus like particles correctly display β-barrel from meningococcal factor H-binding protein through genetic fusion

**DOI:** 10.1371/journal.pone.0273322

**Published:** 2022-09-16

**Authors:** Luigia Cappelli, Paolo Cinelli, Fabiola Giusti, Ilaria Ferlenghi, Sabrina Utrio-Lanfaloni, Newton Wahome, Matthew James Bottomley, Domenico Maione, Roberta Cozzi

**Affiliations:** 1 University of Bologna, Bologna, Italy; 2 GSK, Siena, Italy; 3 GSK, Rockville, Maryland, United States of America; New York State Department of Health, UNITED STATES

## Abstract

Recombinant protein-based vaccines are a valid and safer alternative to traditional vaccines based on live-attenuated or killed pathogens. However, the immune response of subunit vaccines is generally lower compared to that elicited by traditional vaccines and usually requires the use of adjuvants. The use of self-assembling protein nanoparticles, as a platform for vaccine antigen presentation, is emerging as a promising approach to enhance the production of protective and functional antibodies. In this work we demonstrated the successful repetitive antigen display of the C-terminal β-barrel domain of factor H binding protein, derived from serogroup B Meningococcus on the surface of different self-assembling nanoparticles using genetic fusion. Six nanoparticle scaffolds were tested, including virus-like particles with different sizes, geometries, and physicochemical properties. Combining computational and structure-based rational design we were able generate antigen-fused scaffolds that closely aligned with three-dimensional structure predictions. The chimeric nanoparticles were produced as recombinant proteins in *Escherichia coli* and evaluated for solubility, stability, self-assembly, and antigen accessibility using a variety of biophysical methods. Several scaffolds were identified as being suitable for genetic fusion with the β-barrel from fHbp, including ferritin, a *de novo* designed aldolase from *Thermotoga maritima*, encapsulin, CP3 phage coat protein, and the Hepatitis B core antigen. In conclusion, a systematic screening of self-assembling nanoparticles has been applied for the repetitive surface display of a vaccine antigen. This work demonstrates the capacity of rational structure-based design to develop new chimeric nanoparticles and describes a strategy that can be utilized to discover new nanoparticle-based approaches in the search for vaccines against bacterial pathogens.

## Introduction

Self-assembling protein nanoparticles (NPs) have the intrinsic ability to assemble spontaneously into highly ordered and symmetric molecules [1]. In nature protein NPs are involved in many physiological mechanisms and they are ubiquitously expressed in eukaryotic, bacterial, and viral organisms [2, 3]. In contrast, virus-like particles (VLPs) are protein nanoparticles made *in vitro* by the capsid structural proteins of a virus which are able to self-assemble into symmetric supramolecular architectures that mimic the repetitive surface structure of the natural virus but lack encapsulated genetic material [4]. The intrinsic stability, the internal empty spaces, and the symmetric shape make them useful tools for many pharmaceutical applications [5]. During the years, NPs and VLPs attracted a lot of attention in vaccinology as antigens *per se* or as antigen display platforms [6–11]. In particular, VLPs and NPs can potentially allow to overcome the intrinsic low immunogenicity of monomeric protein antigens for the production of effective recombinant protein-based vaccines [1]. Several vaccines based on VLPs have been successfully developed and marketed, including those to protect against Human Papilloma Virus, Hepatitis B Virus, and malaria [12–16]. Currently, several other chimeric VLPs and NPs are under investigation both in preclinical and in clinical studies to fight different viral pathogens like SARS CoV-2, respiratory syncytial virus (RSV), human immunodeficiency virus (HIV), and influenza (flu) virus [17–20]. Moreover, VLPs and NPs are receiving growing interest also for the development of vaccines against bacterial pathogens [21–23]. The possibility to simultaneously expose many copies of target antigen to the immune system and to avidly bind B-cell receptors eliciting higher functional antibody titers makes NPs a cutting-edge technology for vaccine development [11, 24, 25].

Antigen display on the external surface of NPs has been demonstrated using three main different approaches: genetic fusion, protein ligation systems and chemical conjugation [26]. The latter approach is based on the chemical treatment of both NP and antigen in order to produce a cross-link between the exposed lysines or cysteines [27]. However, chemical modification could alter the physiochemical properties and the structure of the scaffold or the antigen compromising the stability and functionality of the resulting chimeric NPs. Protein ligation systems ingeniously exploit the ability of two different protein components to form a site-specific irreversible isopeptide bond. Fusing the antigen to the scaffold it is possible to obtain an *in vitro* assembled chimeric NP [28–30]. The major drawback of this approaches is the production process, in which multiple steps are required to separately produce the single components and subsequently *in vitro* assemble chimeric NPs. A valid alternative is represented by genetic fusion which requires strong structure-based design knowledge. In this case the gene fragment encoding the antigen of interest is fused through a linker to the NP gene in the expression vector, allowing production of a chimeric molecule as a single polypeptide chain and consequently a simple production process.

The genetic fusion approach has been investigated in the present work to achieve the display of a protein antigen on the surface of various protein NPs and VLPs. The selected scaffolds are characterized by different numbers of subunits, size, and geometry, summarized in Table 1. The smallest NP tested was the ferritin particle from *Helicobacter pylori*, composed by 24 subunits each of which folds into a four-α-helix bundle with a resulting octahedral geometry [31]. With an increased number of subunits (60) and an icosahedral geometry the encapsulin from *Thermotoga maritima* has also been tested [3, 32]. Both proteins are naturally occurring molecules involved in different biological roles such as iron storage or molecular compartmentalization. Moreover, in the last years some evidences of their applicability as display platforms have been reported [23, 31, 33, 34]. An additional protein NP tested was mI3, a computationally derived scaffold obtained after several cycles of mutations and optimization of *T*. *maritima* trimeric aldolase [35, 36]. In mI3, 60 subunits are linked together by multiple polar interactions that make the icosahedral T1 structure highly stable in extreme conditions of pH and temperature [35]. In order to increase the size of NP tested, also three VLPs: Qβ, AP205 (CP3) and HBcAg were investigated. The first two are the capsid proteins of respectively Qβ and AP205 bacteriophages able to infect *E*. *coli*. Their 180 subunits are structured in icosahedral T3 geometry with a diameter ranging from 25-30nm [36–42]. The last VLP tested was the hepatitis B virus protein capsid HBcAg which with 34 nm of diameter and 240 subunits was the largest VLP tested [22, 43–45]. Recently, the successful use of HBcAg as a scaffold for the display through genetic fusion of two meningococcal antigens (fHbp and NadA) has been reported. Mouse immunogenicity studies showed the ability of these chimeric HBcAg VLPs to raise high levels of binding antibodies, with bactericidal activity when the HBcAg fusions contained NadA [22].

**Table 1:** Summary of protein-based nanoparticles and virus like particles tested in this study.

Protein based nanoparticles							
	Organism	MW (KDa)	PDB code	N° Subunit	MW NP (KDa)	Diameter (nm)	Phase study
**Ferritin**	*H*. *pylori*	15	3BVE	24	360	10	Clinical
**mI3**	*Computationally designed from *T*. *maritima* aldolase	25	5KP9	60	1500	18	Preclinical
**Encapsulin**	*T*. *maritima*	32	3DKT	60	1920	24	Preclinical
**Virus-like particles**							
**CP3**	AP205 bacteriophage	15	5LQP	180	2700	30	Preclinical
**Qβ**	Qβ bacteriophage	16	1QBE	180	2880	30	Clinical
**HBcAg**	Human hepatitis B virus	16	1QGT	240	3840	34	Clinical

*mI3 derives from the optimization of the I03 nanoparticle previously designed by Hsia et al. [35, 36]. The structures of mI3 and I03 have not been reported, consequently for the structural analysis a closely related icosahedral T. maritima particle (see PDB 5KP9) was used as a reference.

The target antigen used herein to screen different NPs and VLPs was the lipoprotein Factor H binding protein (fHbp) from serogroup B Meningococcus (MenB) [46, 47]. fHbp sequences can be classified in three main variants which are generally non-cross-protective. As a highly protective antigen, fHbp is a key component of two different vaccines against MenB, namely 4C [48] MenB (Bexsero) and MenB-fHbp (Trumenba) [49–51]. The fHbp 3D structure is well characterized [47, 52]. The protein has a molecular weight of about 27 KDa and exhibits two β-barrels connected by a short linker [47]. Recently a set of human monoclonal antibodies (mAbs) have been reported to recognize fHbp [53, 54]. In particular, a crystal structure of the complex of fHbp with the bactericidal human mAb called 4B3 has been obtained revealing the presence of conformational and cross-protective epitope [55].

Previous studies have shown that the C-term b-barrel of fHbp harbour the most protective epitopes (e.g. targeted by the cross-protective humAb 4B3 [56] and the cross-reactive humAb 1A12 [57]. In this work, computational and structure-based approaches have been applied to identify self-assembling protein NPs and VLPs which can correctly display the fHbp β-barrel on their surface through genetic fusion, allowing the simplification of the production process. Here the design strategy, the recombinant production in *E*. *coli* and biochemical and structural characterization of each chimera are reported.

## Materials and methods

### Rosetta comparative modelling to predict 3D structure of resulting chimeras

The design and the 3D structure prediction of each chimeric nanoparticle displaying the fHbp β-barrel were obtained with Rosetta’s comparative modelling tool [58]. Throughout this study, the variant 1.1 form of fHbp was used, for which a high-resolution crystal structure was available (PDB entry 3KVD) [59]. To obtain the antigen-decorated scaffold, a model composed of the nanoparticle monomer and the β -barrel domain was manually prepared and aligned to the asymmetric unit of the self-assembling particle [60]. Then, a linker connecting the two portions was conformationally sampled using a fragment-based loop-modelling protocol, with refinement in Rosetta to minimize energetics and resolve clashes [61]. Finally, symmetric constraints were applied to generate the other subunits in order to obtain a 3D structure prediction of the entire nanoparticle. Pymol [62] and ChimeraX [63] software packages were used for structural investigation, molecule visualization and graphical representation.

### Cloning, expression, and purification of recombinant proteins

The genes encoding for designed molecules were synthesized as DNA strings by GeneArt (Thermo Fisher Scientific) optimizing the codon usage for expression in the *E*. *coli* and adding at the gene extremities the appropriate linker for ligation independent cloning. In order to obtain recombinant N- or C- terminally His-tagged proteins, the genes were cloned into pET15b+TEV and pET21b+ (Merck-Sigma) PCR-amplified vectors using the Infusion cloning kit (Takara) following manufacturer instructions. Protein expression was performed using *E*. *coli* BL21(DE3) strain (New England Biolabs). The cells were grown in 500 mL of HTMC media (Glycerol 15 g/L; Yeast Extract 30 g/L; MgSO_4_ x7H_2_O 0.5 g/L; KH_2_PO_4_ 5 g/L; K_2_HPO_4_ 20 g/L; KOH 1 M to pH final 7.35±0.1), under shaking (160 rpm) at 37°C until reaching an optical density OD_600_nm_ of 0.8 followed by induction with 1mM IPTG for 3h at 37°C. Soluble proteins were extracted by sonication for 10 minutes in 20 mM Tris, 150 mM NaCl and EDTA-free protease inhibitors at pH 8 alternating cycles of 30 s pulse and 30 s stop. The first protein purification step was performed with immobilized metal affinity chromatography (IMAC) using Ni-NTA agarose resin (Thermo Fisher Scientific) and an elution buffer containing phosphate buffered saline (PBS) with 350mM of imidazole. Fractions containing the target protein were applied to a size exclusion chromatography (SEC) column (Superdex 200 10/300, GE Healthcare) equilibrated in PBS buffer, with a flow rate of 0.5 mL/min; the NP proteins were collected in the void volume due to their large sizes. SDS-PAGE analysis was performed to check protein purity and the concentration was determined by UV-Vis absorbance at 280_nm_ (Nanodrop device).

#### Negative staining electron microscopy

The electron microscopy analysis was performed loading 5 μl of sample concentrated 20 ng/μL onto a glow discharged copper 300-square mesh grid for 30 s. Blotted the excess, the grid was negatively stained using NanoW for 30 seconds. The samples were analysed using a Tecnai G2 spirit and the images were acquired using a Tvips TemCam-F216 (EM-Menu software).

#### Dot blot

5 μg of proteins were spotted on a nitrocellulose membrane and let adsorb for 10 minutes. The membrane was then blocked with 3% milk in PBS and 0.1% Tween detergent. The binding with primary antibody 4B3 [56] (diluted 1:1000 in 3% milk) was followed for 1h at room temperature (18–26°C) with gentle shaking, then the membrane was washed three times with 10mL of PBS and 0.1% Tween for 5 minutes each. The secondary antibody conjugated with horseradish peroxidase (HRP) was then added (diluted 1:1000) in 3% milk to the membrane for 1h. Three additional wash steps were performed, as above, before adding the chromogenic substrate 4-chloro-1-naphthol in order to acquire the signal using GelDoc XR+ imaging system.

#### Surface plasmon resonance

The capability of human mAb 4B3 [56] to recognize the fHbp1.1 β-barrel nanoparticles was assessed by SPR analysis using the Single Cycle Kinetics method [64]. mAb was diluted to a concentration of 5 μg/mL with running buffer HBS-EP+ (0.01 M HEPES, 0.15 M NaCl, 0.003 M EDTA and 0.05% v/v Surfactant P20) and captured on the surface of a CM5 sensor chip coated with a secondary anti-human IgG Fc. Increasing concentrations (1.25 nM, 2.5 nM, 5 nM, 10 nM, 20 nM) of each analyte were injected for 60 s on the surface of the sensor chip. After the last injection, dissociation of the protein was followed for 1500 s. After each cycle, the sensor chip was regenerated using 3 M MgCl_2_. The sensorgram, a plot of response (measured in *Resonance Units* [RU]) against time (measured in seconds [s]), was used to monitor the interaction. The response is directly proportional to the concentration of biomolecules on the surface. The sensorgrams resulted from the blank subtraction, based on the captured mAb but with injections of buffer instead of samples. Capture adjustment was applied to correct sample responses for variations in the levels of captured mAb between cycles by dividing the sample response with the response for captured ligand. Adjusted response levels are expressed as sample response divided by capture level.

## Results

### Rosetta comparative modelling for the structural assessment of chimeric NPs

An *in-silico* analysis of the structures and the symmetry of each NP for the correct design of the NP-antigen chimeras was performed. Inspection of the subsequent NP models (Table 1) allowed the identification of candidate sites for the design of chimeric NPs. In particular, the C-terminal portion of each NP is directed inside the particle scaffold (with the only exception of HBcAg) or involved in the interface interactions needed for the particle assembly as reported in literature. In contrast, the N-terminal portion is exposed on the NP surface and is therefore considered more suitable for antigen display, potentially achievable by genetic engineering [6, 32, 35, 38, 65, 66]. In the case of HBcAg and encapsulin, a relatively long surface-exposed loop was also identified and selected to be tested as an additional antigen insertion point [67].

**Fig 1:**
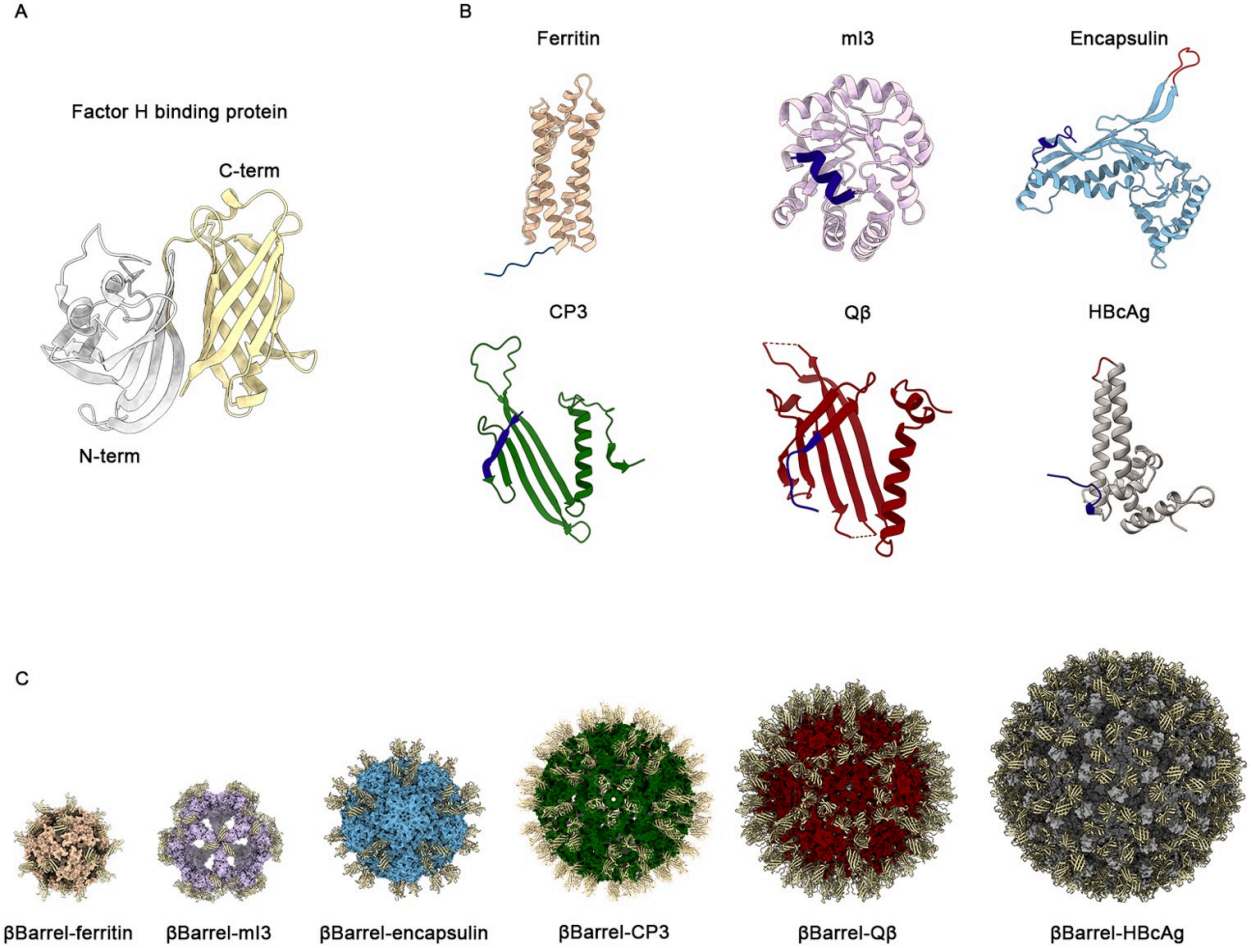
Structural analysis and in silico design of chimeric NPs. (A) Cartoon representation of 3D structure of fHbp antigen (pdb code 3KVD). In grey it is reported the N-terminal domain (residues 1–118) while in yellow it is shown the C-terminal βbarrel domain used in this work (residues 119–249). (B) Cartoon representation of the monomeric structure of each tested NPs. Engineerable sites explored for the genetic fusion of the antigen are highlighted: the N terminus in dark blue and the exposed loops in red. (C) Cartoon of predicted 3D models of each chimera obtained with Rosetta homology modelling. The βbarrell exposed was represented in yellow. Images were obtained with ChimeraX (panel A, C) and Pymol (panel B).

The test antigen selected for this study was the well-characterized fHbp antigen, specifically variant 1.1, from MenB. While diverse mAbs from mice and humans have been shown to target epitopes in both the N- and C-terminal β-barrel domains of fHbp, recent structural studies have localized the epitopes of potent cross-protective human mAbs 1A12 [57] and 4B3 [56] on the C-terminal domain. However, in the total set of human mAbs analysed, such cross-reactive mAbs were relatively rare (approximately only 10%) [53, 55]. Therefore, to promote a beneficial immuno-focusing effect, the C-terminal β-barrel domain of fHbp (residues 119–249 PDB code 3KVD), was selected for display on the surface of each NP (Fig 1A).

Based on the structural analyses above, the gene encoding for the antigen fragment was fused at the N terminus of each NP gene, spaced by a glycine-serine linker [68, 69] and in the gene sequence of exposed loop of HBcAg) [67] and encapsulin (Fig 1B). For each chimera a 6-His tag was inserted at the N-term of the antigen to allow protein purification and detection (Fig 1B). The only exception was the chimera based on mI3, in which the His-tag was placed at the C terminus of the scaffold spaced by a glycine-serine linker.

In order to analyse the spatial disposition of the antigen on the NP surface, a structural prediction of the symmetric assembly was performed with Rosetta comparative modelling [70]. The chimeric sequences were threaded onto template structures consisting of both antigen and NPs, and an energetic analysis of these models was performed to ensure the absence of steric clashes, while assessing the conformational feasibility of repetitively displaying the fHbp βbarrel on the NP surface in a symmetric manner (Fig 1C and S1 Fig).

### Correctly assembled NPs were detected for all tested molecules, except Qβ

**Fig 2:**
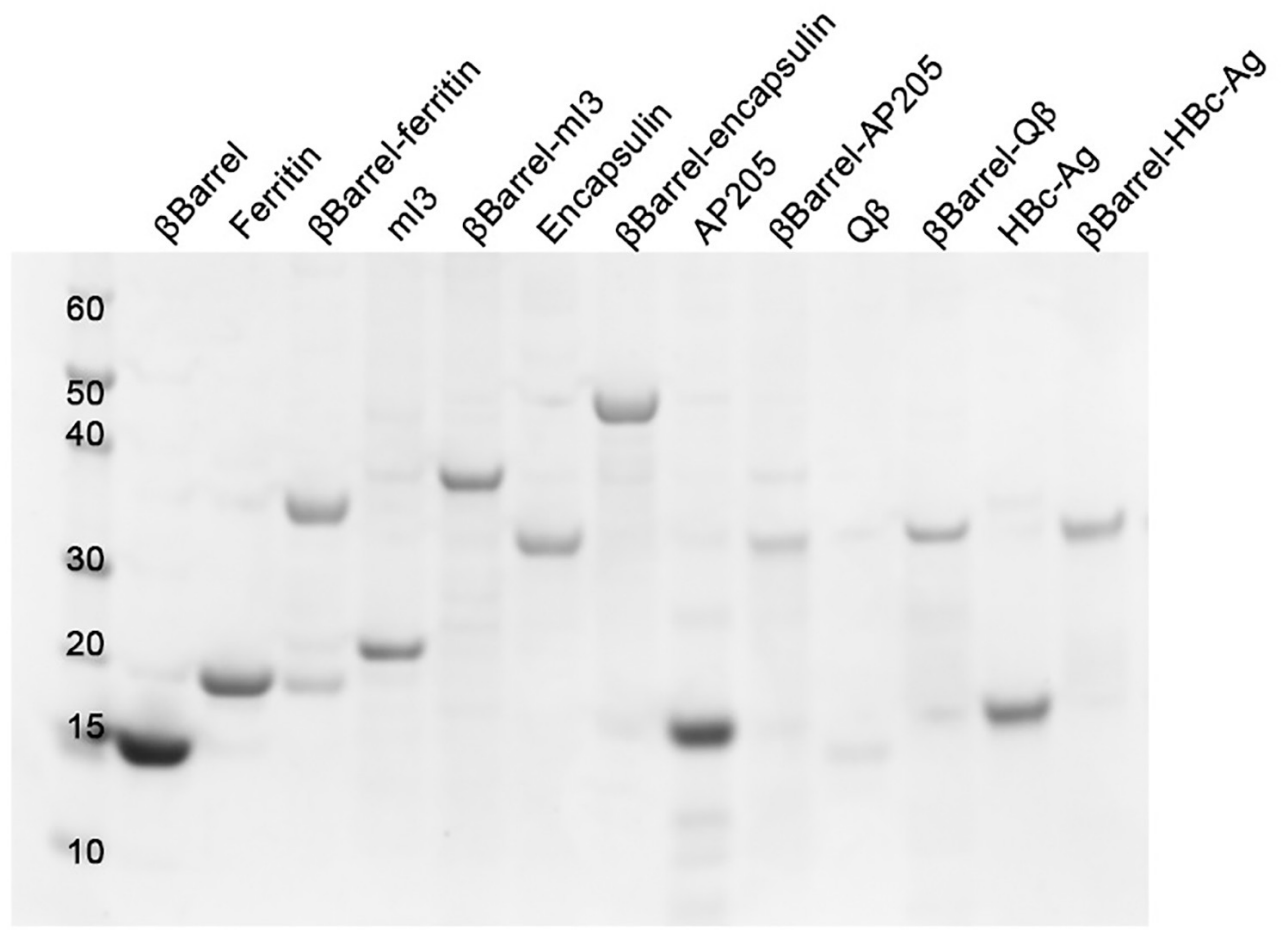
SDS-PAGE analysis of purified monomeric antigen, naked and chimeric NPs after SEC purification, performed under denaturing conditions and stained with Coomassie blue. First lane reports the molecular weight marker expressed in KDa. Theoretical molecular weights of each sample: βbarrel 14KDa, Ferritin 21KDa, βbarrel-Ferritin 34,8KDa, mI3 23,6 KDa, βbarrel-mI3 37,3KDa, encapsulin 32,1 KDa, βbarrel-encapsulin 45,9 KDa, CP3 15,2 KDa, βbarrel-CP3 29,5KDa, Qβ 16,1 KDa, βbarrel-Qβ 29,7KDa, HBcAg 19KDa, βbarrel-HBcAg 32,3 KDa.

**Fig 3:**
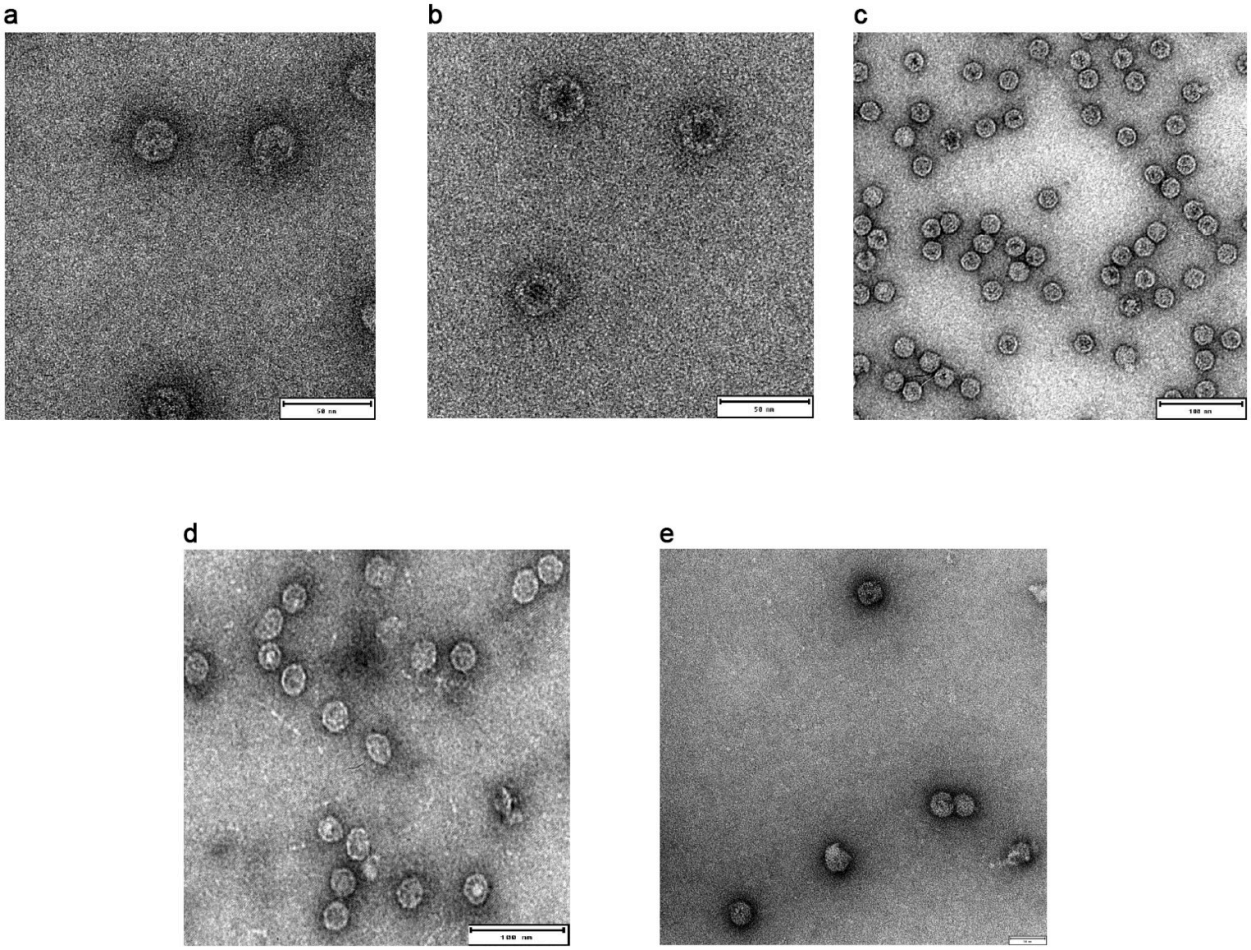
Negative staining transmission electron microscopy (NSTEM) of chimeric NPs displaying βbarrel antigen after SEC purification. Properly assembled particles were detected for (A) βbarrel-Ferritin with a diameter of 25nm (B) βbarrel-mI3 with a diameter of 30nm (C) βbarrel-Encapsulin presents a diameter of 30nm (D) βbarrel-CP3 presents a diameter of 30nm (E) βbarrel-HBcAg with a diameter of 35nm. Scale bars inserted in the pictures correspond to 50nm (A-B-E) and 100nm (C-D).

All designed chimeras resulted to be well expressed and soluble when recombinantly produced in *E*. *coli* BL21(DE3). Correctly assembled NPs were isolated from the soluble fraction after two steps of purification, affinity, and size exclusion chromatography (SEC). The integrity and purity of each sample was assessed with SDS-PAGE analysis in denaturing conditions (Fig 2). Each monomer migrated at the expected molecular weight (MW). Furthermore, the shift in MW, by comparing chimeric constructs with naked NPs, confirmed that the polypeptide of the expected length was produced and that this chimera is not susceptible to protease digestion. The structure of the protein purified by SEC was analysed by negative staining with transmission electron microscopy (TEM). The genetic fusion of the antigen at the N-term of each NP was successful. In fact, all protein-based chimeric NPs resulted in a homogeneous population of correctly assembled NPs with a diameter ranging from 25 nm (βbarrel-ferritin) to 30nm (βbarrel-mI3 and βbarrel-encapsulin) (Fig 3A–3C). For this last construct, we also observed a tendency of NPs to adhere to each other; in fact, NPs completely separated from the others were rare. On the other hand, only two out of the three chimeras based on VLPs were correctly assembled into NPs. In fact, only for βbarrel-CP3 and βbarrel-HBcAg, properly structured NPs were detected, with a diameter of 30 and 35nm respectively (Fig 3E–3D). Despite several attempts, βbarrel-Qβ nanoparticles were not obtained as ordered structures, but only aggregated and precipitated proteins were detected in TEM analysis (S2A Fig).

### βbarrel antigen is correctly displayed on NP surface

**Fig 4:**
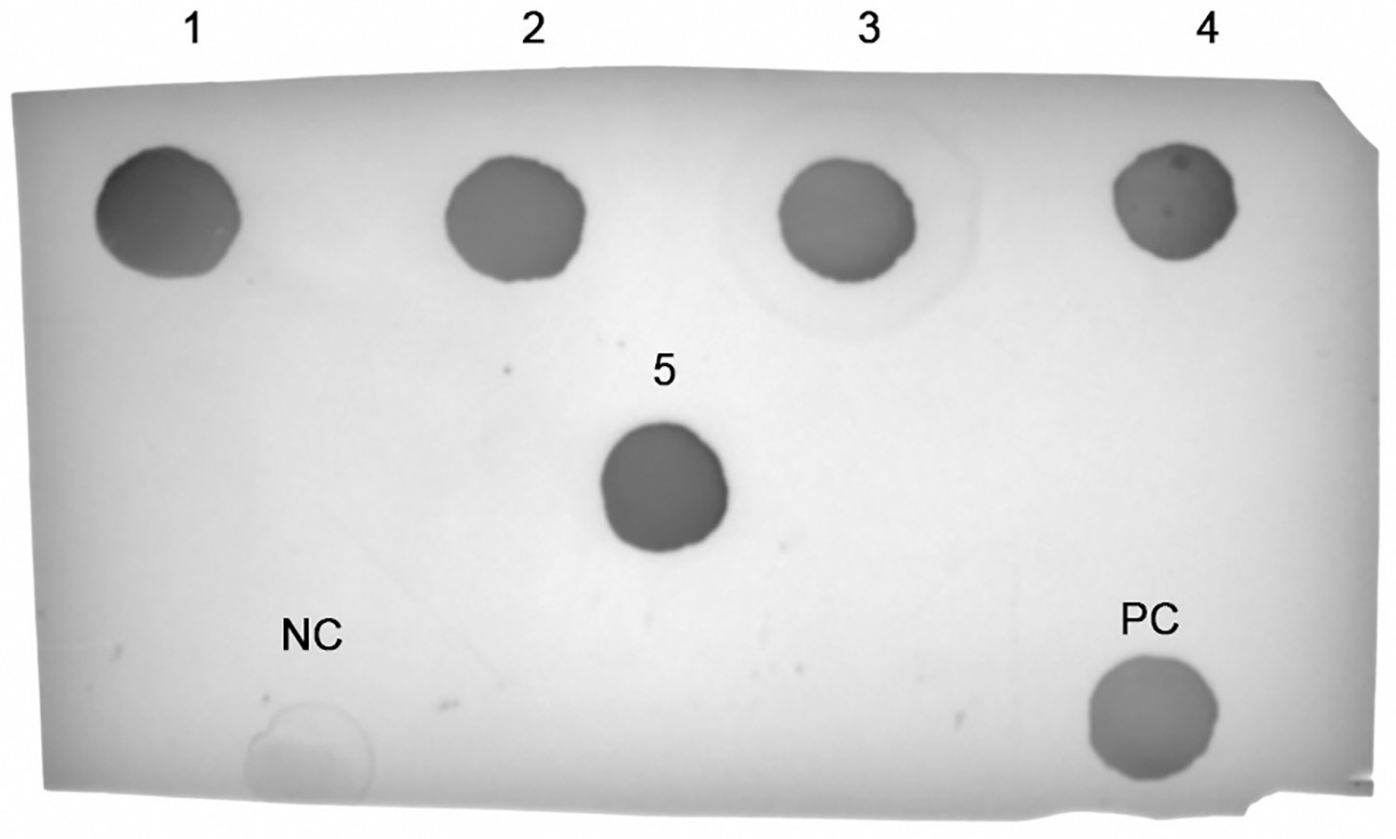
Dot Blot analysis of different chimeric NPs displaying βbarrel for the detection of exposed antigen. (1) βbarrel-ferritin, (2) βbarrel-mI3, (3) βbarrel-Encapsulin, (4) βbarrel-CP3, (5) βbarrel-HBcAg, (NC) Negative control represented by naked ferritin, (PC) Monomeric βbarrel used as positive control.

To further investigate the antigen structure and conformation on the NPs surface, a dot blot assay was performed (Fig 4). To achieve our purpose, the human monoclonal antibody (hmAb) 4B3 able to bind a βbarrel conformational epitope was used [56]. An interaction between 4B3 and the antigen was observed by dot blot assay for monomeric βbarrel used as positive control and for all NPs tested. These data confirm that not only the antigen is present, but it is also correctly structured and appropriately displayed to be accessible for antibody recognition. In accordance with this, no binding was detected between 4B3 and naked ferritin used as negative control.

**Fig 5:**
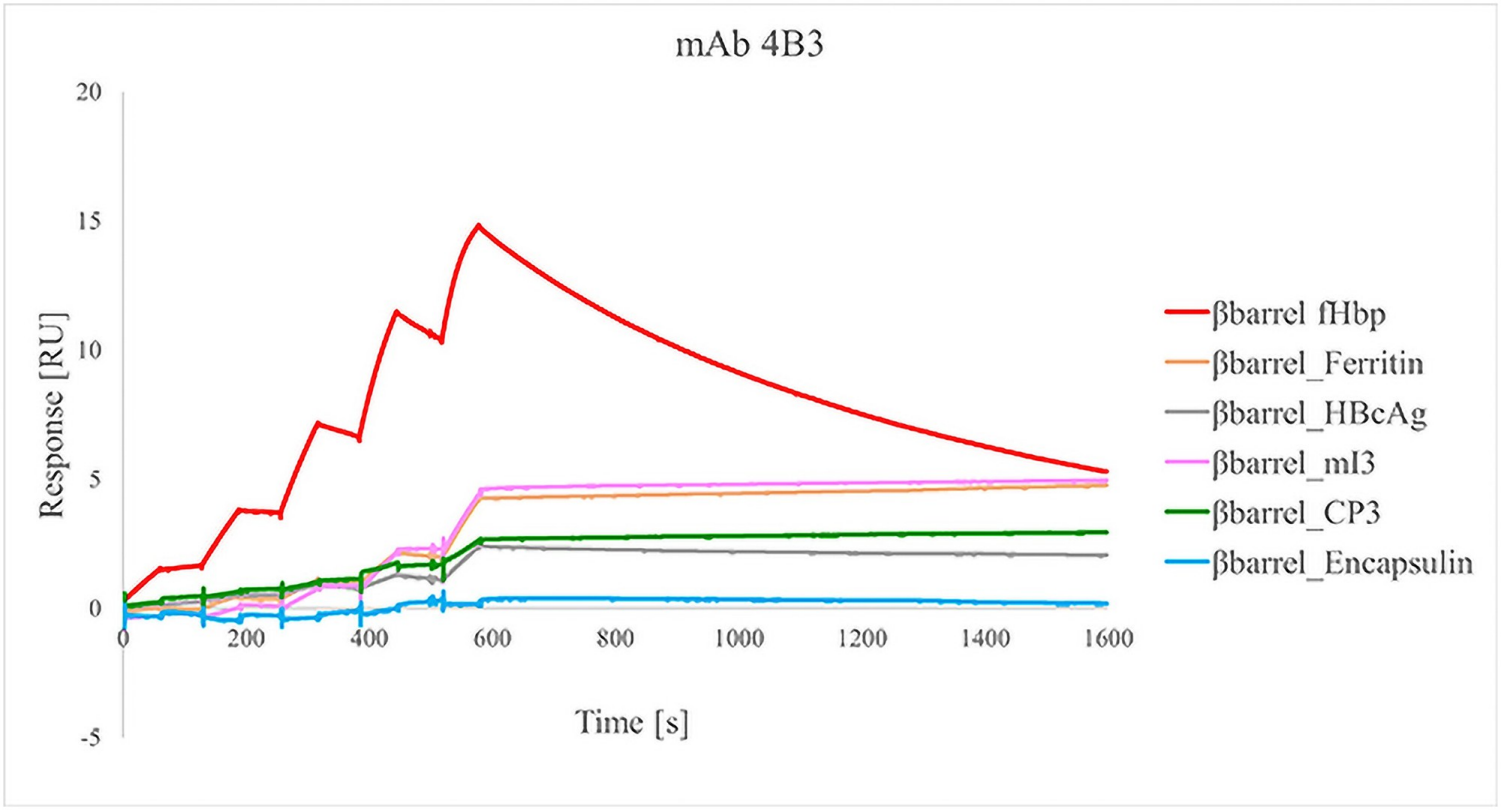
Biacore SPR analysis of monomeric βbarrel and βbarrel-NPs for evaluation of binding avidity with cross-bactericidal 4B3 hmAb. The interaction between the sample and the antibody has been observed during a time frame of 1500s.

Moreover, one of the advantages to use NPs as a scaffold is the possibility to display multiple copies of a target antigen. This results in the improvement of the avidity that is crucial for the induction of potent and long-lasting immune responses [1, 26, 71]. For this reason, we evaluated the avidity of the binding between βbarrel and 4B3 with a surface plasmon resonance (SPR) assay (Fig 5). The hmAb was captured on the surface of a sensor chip and increasing concentrations of the analytes (βbarrel alone and chimeric βbarrel-NPs) were applied (see Materials and methods). The kinetic parameters of the interaction between monomeric βbarrel and 4B3 were evaluated using the Langmuir 1:1 binding model; both association (k_on_) and dissociation (k_off_) constants were measured with a resulting K_D_ of 1,114E^-9^ M ± 0.219 (Fig 5 red line). While, regarding the βbarrel-NPs, the kinetic parameters evaluation was not applicable to all the samples because of the avidity effect. Indeed, during the dissociation phase, the complex formed between mAb, and some βbarrel-NPs did not dissociate over time but remained stable. Notably, although the samples were normalized for protein content, the highest binding level was displayed by monomeric βbarrel. Moreover, we observed an inversely proportional tendency between molecular weight (MW) and RU reached by the molecules. This could be explained by the fact that during the analyte injection, applying a constant flow rate, a higher number of binding events occur with molecules with lower MW because the higher MW molecules move slower over the sensor chip surface. This tendency was common to all chimeras except βbarrel-Encapsulin for which it was impossible to evaluate the binding profile. Its tendency to adhere, highlighted also with TEM, likely masks some epitopes making them unavailable for the binding with the hmAb.

### The insertion of β-barrel in exposed loops led to the formation of inhomogeneous NPs

**Fig 6:**
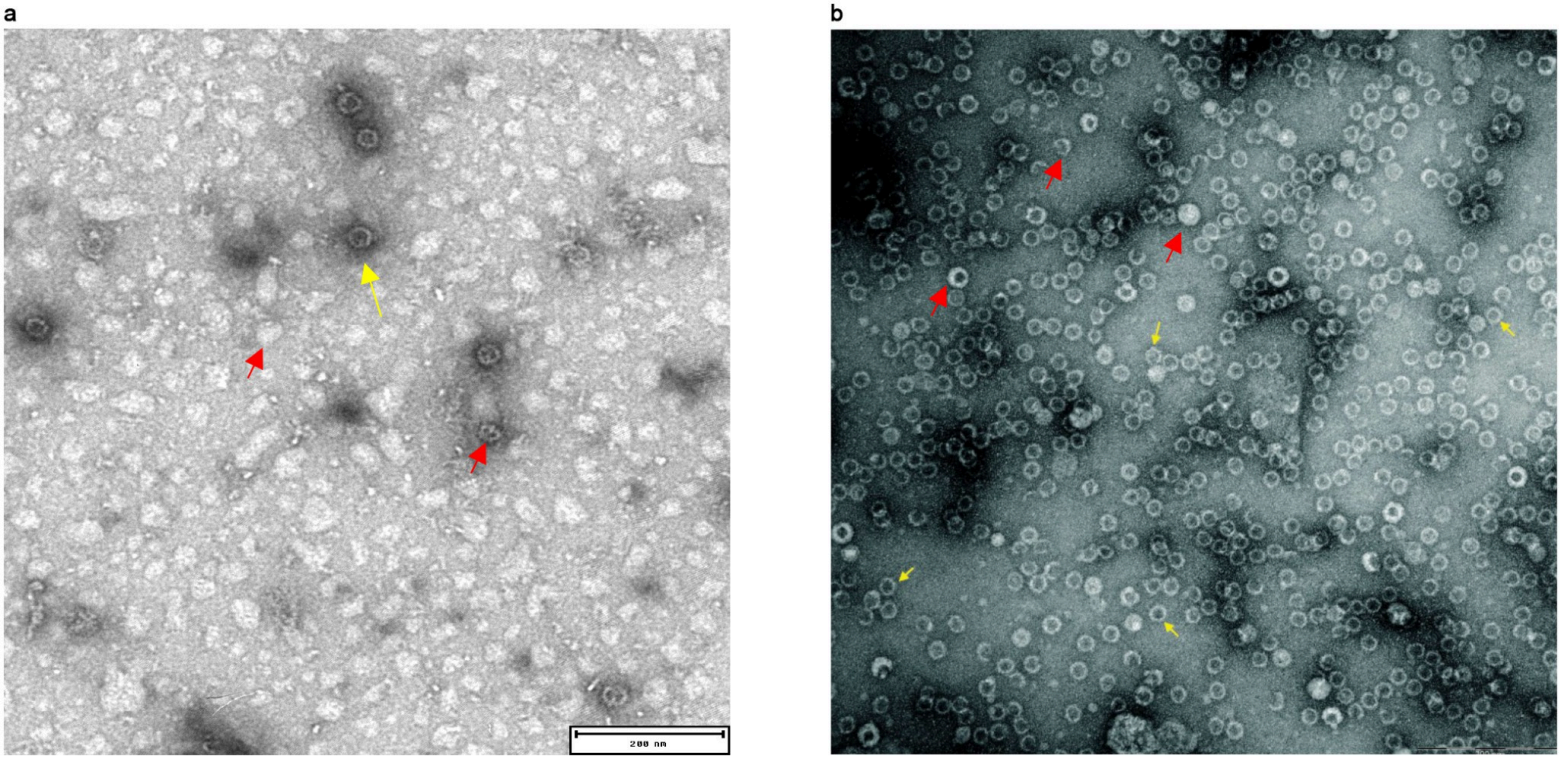
Negative staining transmission electron microscopy (NSTEM) analysis of HBcAg (A) and encapsulin (B) NPs exposing βBarrel in the loop. Yellow arrows indicate correctly assembled NPs. Red arrows indicate incorrectly structured NPs like aggregates, NPs partially structured or NPs with unexpected size or geometry. Scale bars inserted in the pictures correspond to 200nm (A-B).

The display of βbarrel antigen was also tested by engineering an exposed loop of HBcAg and encapsulin NPs. In particular, for HBcAg we exploited the connecting loop of α3 and α4 helices of each monomer. This site is part of the immunodominant B-cell epitope detected from amino acids 74–84 and the loop design with one or two different protein domains has been reported previously [67, 72]. Therefore, the βbarrel was inserted between residues 79 and 80. Although the chimeric protein was produced at high level in soluble form in *E*. *coli* only a minimal portion of proteins were correctly assembled. In fact, TEM analysis revealed that the majority of proteins were aggregated or only partially structured (Fig 6). An analogous result was obtained by engineering a selected loop of encapsulin. The analysis of the crystal structure of naked encapsulin [32] revealed that the loop from residues 58–64 is flexible and is well exposed on the surface of the NP. For this reason, the chimeric construct was generated by inserting βbarrel between residues 58 and 64 replacing the original sequence including amino acids 59–63. The recombinant production of the chimeric molecules allowed us to obtain only heterogeneous samples with particles differing by size and shape (Fig 6). Moreover, further production attempts failed, and the chimeric protein was detected only as a monomer (S2B Fig).

## Discussion

Protein-based vaccines are safer than traditional vaccine preparations based on live-attenuated, or killed pathogens [73]. However, the major drawback is the lower immune response induced by single purified proteins or oligo/polysaccharide components, and consequently fusion proteins with increased size, multiple doses and adjuvants are often required in order to achieve sufficient immunogenicity [74–76]. Moreover, the low efficiency of vaccine antigens could be due to immunologically subdominant but protective epitopes [77]. To overcome these issues, self-assembling protein NPs are now widely explored in vaccinology as scaffolds for antigen display [1]. In fact, the use of a larger scaffold combined with the multicopy display of target antigen allows an efficient activation of B-cell receptors and longer retention in lymphoid follicles [26]. The result is the potential induction of a potent B- and T-cell response. Currently, numerous chimeric NPs are under investigation in preclinical and clinical research world-wide [44, 78, 79]. In these studies, several different self-assembling protein NPs and VLPs have been decorated with protein antigens of interest through genetic fusion, protein ligation or chemical conjugation [6, 37, 80]. In literature are present several examples of viral antigens or small bacterial epitopes displayed on the surface of NP scaffold through genetic fusion [8, 9, 81, 82]. Only few examples of bacterial antigens displayed on NPs have been reported so far [21, 22]. Compared to other systems, the genetic fusion approach has the enormous advantage to allow the generation of the final nanoparticle by producing a single recombinant protein. However, it is essential that both scaffold and antigen preserve their correct structure after the fusion [83, 84]. This could be particularly challenging in the case of large and bulky antigens as well as multimeric proteins [1]. However, genetic fusion remains the more straightforward approach to produce chimeric NP displaying the antigen of interest. In the present work the feasibility to use genetic fusion for the display of a structured protein antigen on the surface of six different NPs has been investigated. Immuno-focusing by displaying multiple copies of a key epitope on a nanoparticle was previously demonstrated [85]. In this work, βbarrel of fHbp v. 1.1, containing most of the cross-reactive epitopes, has been fused to ferritin, mI3, encapsulin, CP3, Qβ and HBcAg NPs. All these molecules are characterized by different structure, size, shape, and number of subunits. The structural analysis allowed to identify potential sites for the insertion of the antigen. The N-terminus of each molecule resulted to be a region suitable for foreign protein insertion and in the case of encapsulin and HBcAg an exposed loop was also identified as potential insertion site. However, the loop design led the production of inhomogeneous samples containing aggregates and partially formed assemblies. Conceivably, the design at the level of amino acidic sequence of the scaffold interfered with the correct assembly of the NP. This result is in accordance with the recently published work of Aston-Deaville et al., which reports the potential disruption of HBcAg 3D structure following the loop engineering with βbarrel antigen [22]. However, in addition to their data, here we reported the possibility to successfully engineer also the HBcAg N-term with βbarrel. In fact, the genetic fusion of the antigen at the N-term of each scaffold allowed the production of homogeneous and well-structured molecules with the only exception of Qβ VLP. Despite several attempts, Qβ VLPs displaying fHbp βbarrel were not obtained, indicating that the genetic fusion of a protein antigen to the N-terminus of Qβ VLP interfered with the assembly. In accordance, the approach reported in literature for the decoration of Qβ VLP is the chemical conjugation of small protein peptides. On the other hand, all the remaining scaffolds were able to correctly display the βbarrel obtaining NPs with a diameter ranging from 25 to 35 nm. SDS-PAGE analysis confirmed the presence of the antigen in each chimera and dot blot as well as SPR analysis, using functional 4B3 mAb, suggested that the displayed βbarrel was properly folded. However, the SPR analysis revealed that the multicopy display of βbarrel increased the binding avidity and it was impossible to determine the kinetic parameters of the binding. In fact, using self-assembling NPs as scaffold, from 24 (ferritin) to 240 (HBcAg) copies of target antigen were displayed simultaneously on the same molecule. Another advantage of using NPs as scaffold is their intrinsic stability that makes them stable in different conditions such as high temperatures, extreme pH, and presence of denaturants [35, 36, 86]. The thermal stability of each chimeric NPs produced was investigated to understand if the fusion destabilizes the βbarrel or the NP structure.

In conclusion, these data showed that ferritin, mI3, encapsulin, CP3 and HBcAg can be engineered through genetic fusion for the display of fHbp βbarrel, a well-folded protein domain of approximately 15kDa. Chimeric NPs can be easily produced using a standard bacterial expression system and purified as His-tagged proteins. Our data indicate that the design of an internal exposed loop of the NP scaffold is more challenging. In the two cases tested herein, it led to the disruption of the NP structure and the formation of heterogeneous samples. In contrast, the exploitation of flexible and exposed N-terminal regions preserved NP structure and correctly exposed the antigen. Although the choice of the best scaffold may be dependent on the antigen displayed, the identification of five different NPs that can potentially accept genetic fusion at the N-terminus represents a template approach to design and produce new chimeric molecules for both vaccine and drug development. Finally, this work represents a starting point to perform an *in vivo* study of all these molecules to better elucidate the contribution of antigen copy number, size, shape, and geometry in enhancing the immune response and to further investigate NPs mode of action.

## Supporting information

S1 Fig Predicted 3D model of Encapsulin (A) and HBcAg (B) NPs displaying βbarrel into an exposed loop. (PNG)

S2 Fig TEM analysis of unstructured NPs. (A) βbarrel-Qbeta (B) βbarrel-encapsulin. Only aggregates or monomers were detected after affinity and size exclusion chromatography.
(TIF)

S1 Raw images (PDF)
