# Supplementary figures and images for: Self-assembling protein nanoparticles and virus like particles correctly display β-barrel from meningococcal factor H-binding protein through genetic fusion
Source: PLoS One. 2022 Sep 16;17(9):e0273322. doi: 10.1371/journal.pone.0273322 (PMC9480994; doi:10.1371/journal.pone.0273322)

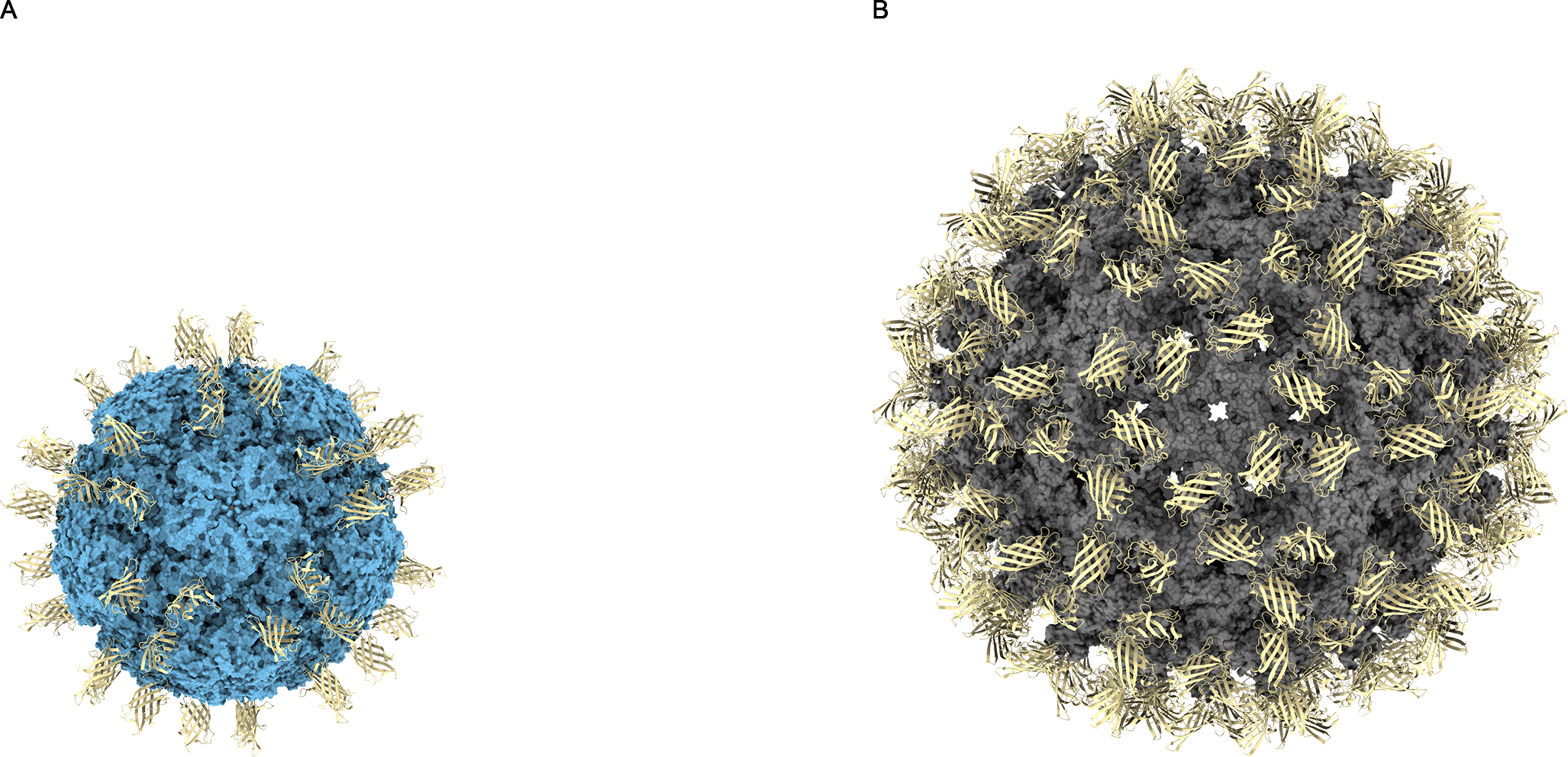

Supplement: S1 Fig — (PNG) [file pone.0273322.s001.png]

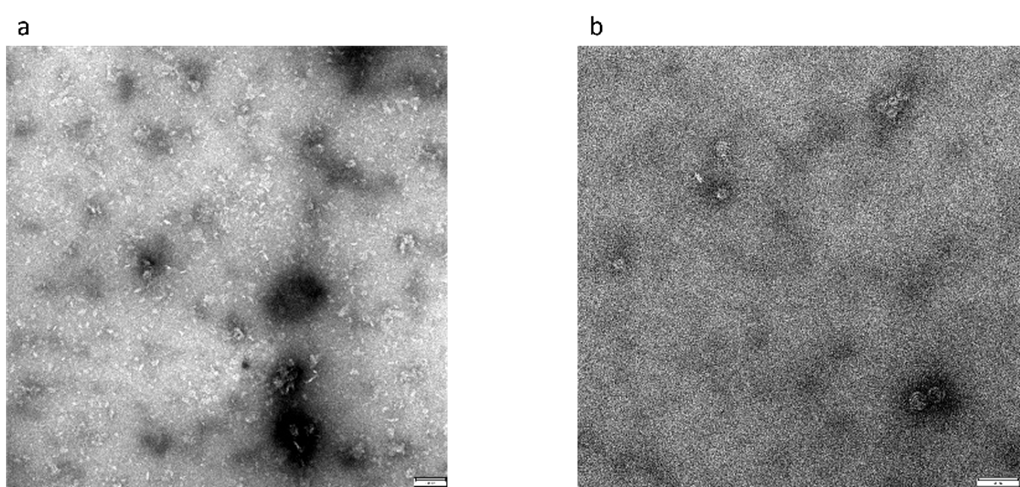

Supplement: S2 Fig — (A) βbarrel-Qbeta (B) βbarrel-encapsulin. Only aggregates or monomers were detected after affinity and size exclusion chromatography. (TIF) [file pone.0273322.s002.tif]
