# Supplementary material for: Self-assembling protein nanoparticles and virus like particles correctly display β-barrel from meningococcal factor H-binding protein through genetic fusion
Source: PLoS One. 2022 Sep 16;17(9):e0273322. doi: 10.1371/journal.pone.0273322 (PMC9480994; doi:10.1371/journal.pone.0273322)
Supplement: S1 Raw images — (PDF) [file pone.0273322.s003.pdf]

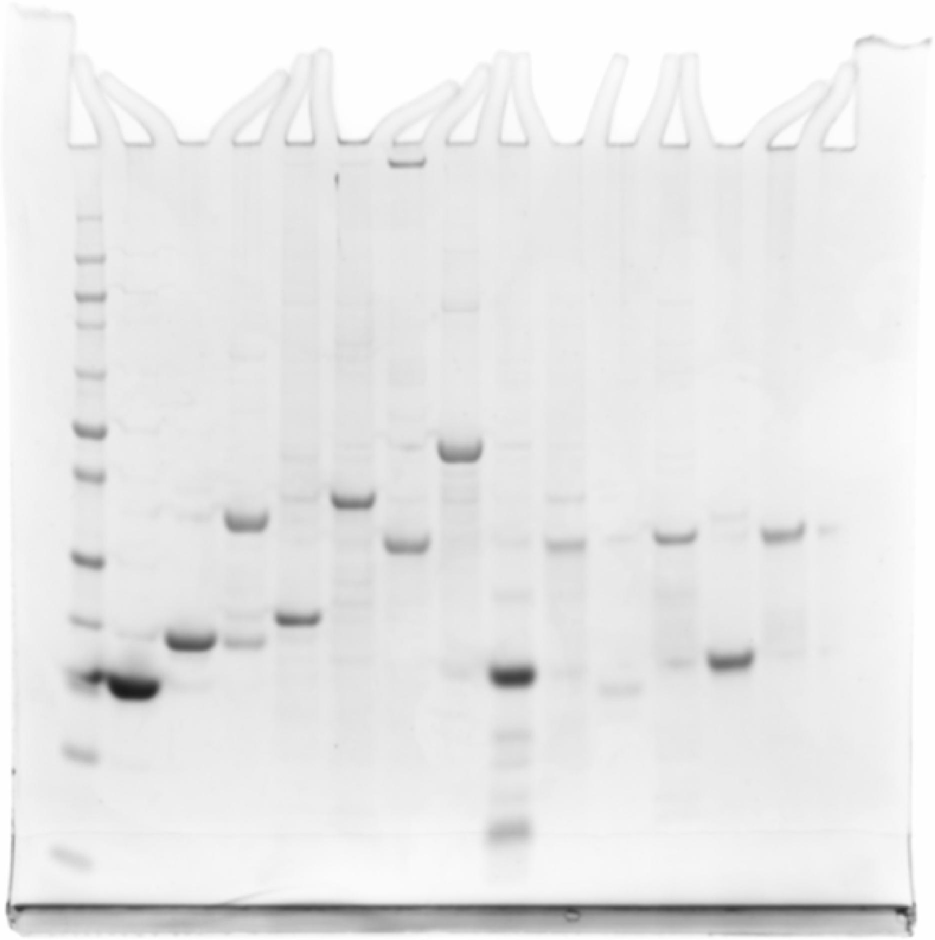

SDS-PAGE loading order: Marker, βBarrel, Ferritin, βBarrel_Ferritin, mI3, βBarrel_mI3, Encapsulin, β Barrel_Encapsulin, AP205, βBarrel_AP205, Qbeta, βBarrel_Qbeta, HBcAg, βBarrel_HBcAg.
Acquired using ImageLab software from BioRad.
From this image, figure 2 was generated.

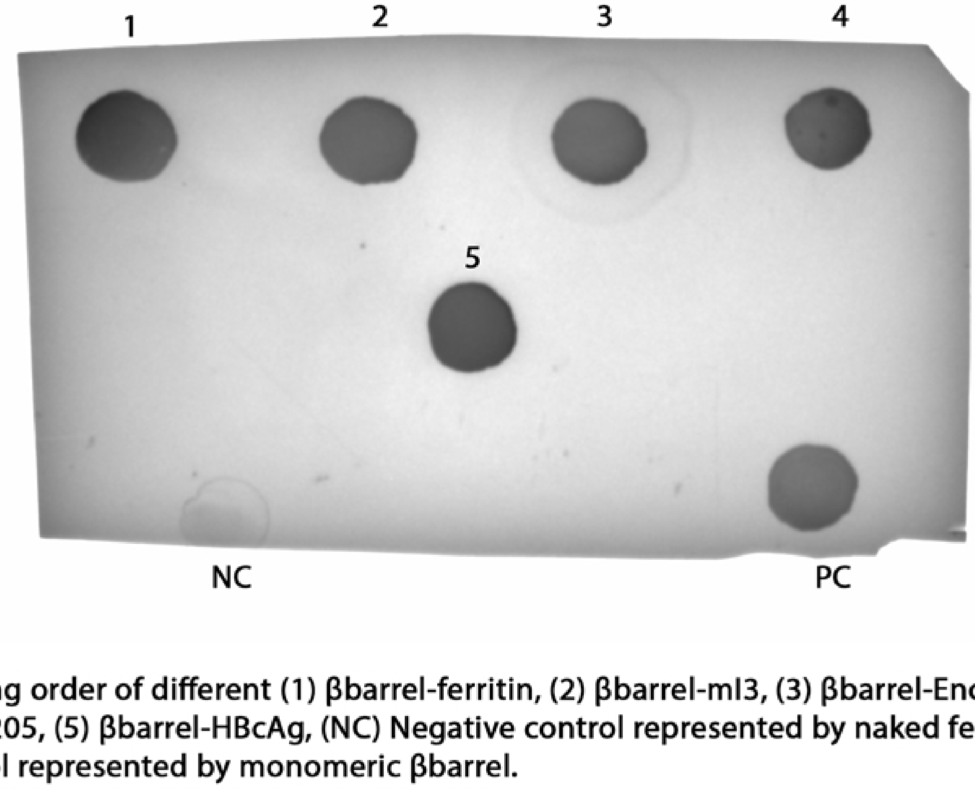

Dot Blot loading order of different (1) βbarrel-ferritin, (2) βbarrel-mI3, (3) βbarrel-Encapsulin, (4) βbarrel-AP205, (5) βbarrel-HBcAg, (NC) Negative control represented by naked ferritin, (PC) Positive control represented by monomeric βbarrel.
Acquired using ImageLab from BioRad.
From this image, figure 4 was generated
